# Editorial: Translating machine learning innovations into clinical practice

**DOI:** 10.3389/fmed.2026.2003342

**Published:** 2026-10-26

**Authors:** Jiawen Deng, Fangwen Zhou, Kiyan Heybati, Anthony Bozzo

**Affiliations:** 1 Division of General Surgery, University of British Columbia, Vancouver, BC, Canada; 2 Department of Biomedical and Molecular Sciences, Faculty of Health Sciences, Queen’s University, Kingston, ON, Canada; 3 Department of Internal Medicine, Mayo Clinic, Rochester, MN, United States; 4 Division of Orthopaedic Surgery, McGill University, Montréal, QC, Canada

**Keywords:** artificial intelligence, external validation, knowledge translation, machine-learning, model generalisation

The medical literature now holds tens of thousands of clinical machine-learning (ML) studies, with many claiming to develop models that could enable individualised, precision medicine. Despite this, relatively few ML models have actually entered routine use at the bedside, and individual deployments are still reported as significant achievements. This discrepancy is often described as a “translational lag,” implying a temporary delay that better engineering, infrastructure, or regulation will eventually close. That framing is perhaps too generous. In many studies, translation is not simply delayed but never incorporated into the research pathway, with model development ending at retrospective validation even when the stated objective is to improve real-world clinical care.

Translation from model development to clinical applications require progression through several distinct stages. After internal validation, models intended for clinical use typically undergo external validation, evaluation on live clinical data via shadow testing, prospective assessment of their impact on patient outcomes through trials, clinical deployment, and continued post-deployment monitoring and updates (Figure 1) (1). Current evidence in the literature becomes progressively thinner along this translation pathway. By 2024, nearly 250,000 publications describing new clinical prediction models had been reported (2). Yet, external validation was reported in fewer than 1% of models among a sample of 10,462 ML algorithms captured across 220 systematic reviews (3). At the point of prospective testing, a 2024 scoping review found only 86 randomised controlled trials evaluating clinical ML systems, with a predominance of single-centre studies and outcomes focused on diagnostic performance rather than patient benefit (4). At the point of clinical deployment, a 2025 systematic review examining real-world implementations of prognostic prediction models identified only 37 publications describing 56 models being used in practice (5). Although these reviews differ in scope and methodology, they consistently show that the development of clinical ML models substantially outpaces evidence of their clinical application.

**Figure 1:**
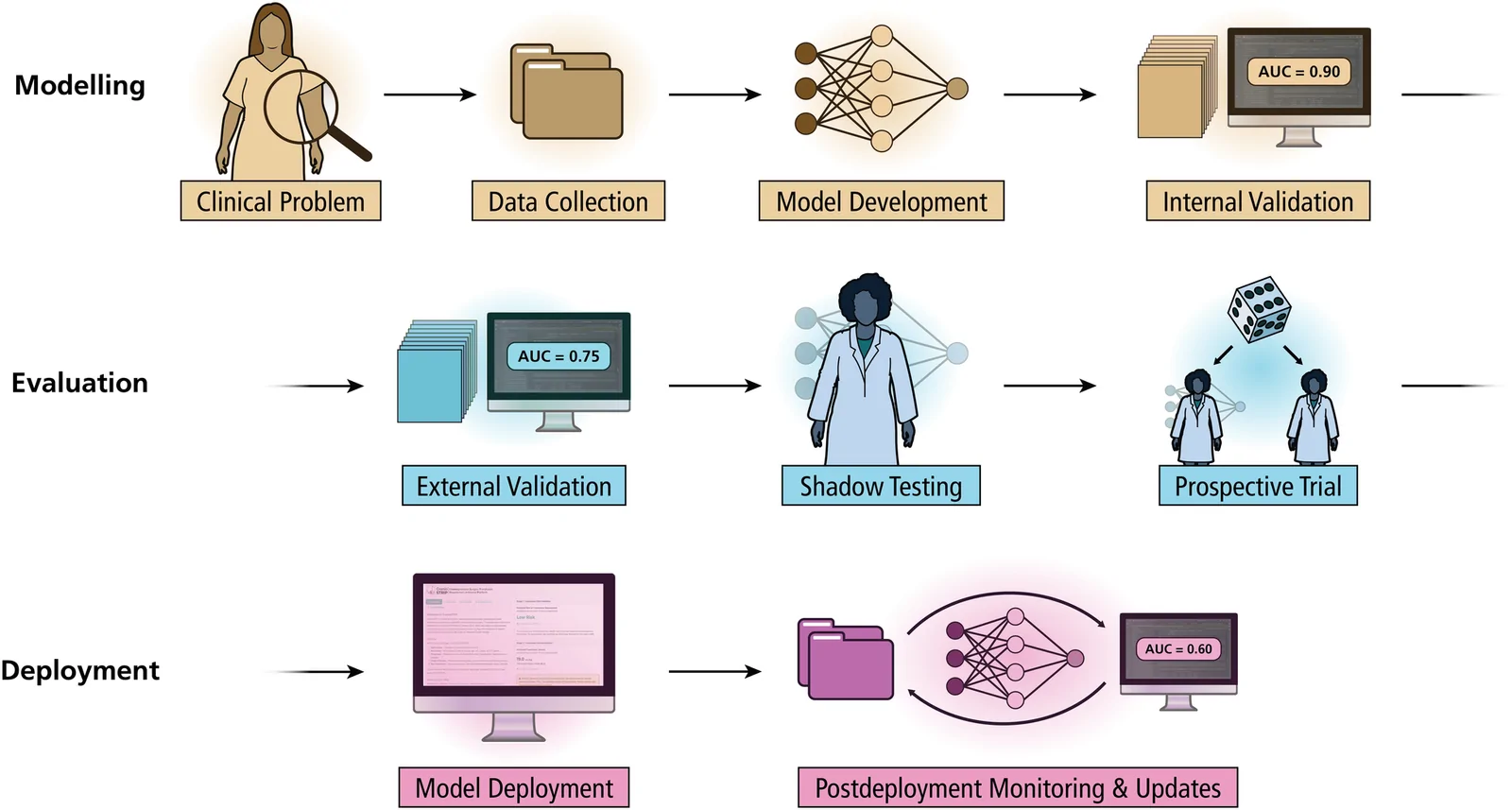
Stages of clinical machine-learning model development, evaluation, and deployment. AUC, area under the curve (of the receiver operating characteristic curve).

This Research Topic aimed to fill this gap in the literature by aggregating contributions that examined the work required to move a model from computer-to-bedside. The six included articles address different points along the model development and translation pathways, beginning with the selection of clinically meaningful problems and the intended role of ML in clinical decisionmaking.

While ML models should ideally be designed to facilitate specific decisions at key inflection points in patient care, many studies instead select their outcomes based on convenience and availability in administrative or electronic health record datasets. This can lead to predictions with no actionable steps to follow and no tangible benefits to patients, leaving little motivation to advance these models to clinical testing. The perspective article by Vinnakota et al. highlights this issue and argues that investigators should consider not simply whether an outcome can be predicted, but whether the prediction resolves a meaningful clinical uncertainty and can lead to an actionable change in management. Mo et al. considers ML in a different context by using it to support assessment of research trustworthiness when conducting systematic reviews, with algorithmic outputs used to flag potentially unreliable studies for expert review rather than replace human judgement. Although the two articles discuss different applications, both illustrate the need to define the intended role of ML models within an existing decision-making process. For clinical models, investigators must therefore specify where predictions should enter the care pathway, which decisions they are expected to inform, and whether they should supplement or replace the role of clinicians.

Beyond selecting a clinically useful target, translation also depends on whether model performance observed during initial internal validation can generalise to external datasets. Two articles in this Research Topic, by Li et al. and Cao et al., largely follow conventional model development pathways but extend their evaluation to include external validation. Their inclusion is notable precisely because this remains uncommon in the literature. External validation provides evidence that model performance is not confined to the derivation dataset and can identify deterioration in performance when models are applied to different patient populations or clinical settings (1).

However, external validation is still performed retrospectively and does not test whether a model can function within real-world clinical environments. Important questions, such as whether variables required by models are available at the point of care, whether predictions can be delivered in an interpretable form, or whether clinicians will incorporate predictions into clinical decisionmaking, can only be answered when investigators study the setting in which models are expected to operate (6). Klooster et al. examined these implementation questions through stakeholder perspectives on predictive models for diabetic foot complications and identified concerns surrounding workflow integration, presentation of predictions, clinical responsibility, and alert burden. Xie et al. similarly examined pharmacists' adoption of AI-based clinical decision support tools and found that their adoption depended not only on perceived usefulness but also on trust as well as organisational and technical support. These considerations are often deferred until after a model has been developed, despite being key to determining whether model deployment would be feasible.

It is notable that most of the articles in this Research Topic did not address the later stages of model evaluation and deployment, such as shadow testing or prospective trials. This does not diminish the impact of contributions included in this Topic, but rather reflects the broader gap in the literature described earlier. The reasons for this persistent gap is likely practical; whereas retrospective validation can be completed using existing datasets within conventional research timelines, shadow testing and prospective studies would require substantially greater coordination and resources (7). At minimum, investigators would need to work with healthcare institutions to integrate models into existing clinical systems, obtain the necessary approvals, and maintain these systems throughout evaluation (8). Such studies may also reveal problems that were not apparent during model development, as shown by Klooster et al. and Xie et al. The prospect of uncovering these problems may further discourage investigators from pursuing prospective study designs. However, without the courage to face and resolve these problems, clinical ML models would remain expensive statistical exercises rather than the paradigm-shifting tools for individualised and precision medicine that they are frequently boasted to be.

Translation also does not end with successful deployment. Changes in patient populations, clinical practice, and data Research Topic can lead to deterioration in model performance over time. Healthcare institutions must therefore establish clear responsibilities for postdeployment surveillance, including regular assessment of model calibration, data drift, and performance across clinically relevant patient subgroups. When deterioration is detected, predefined protocols should guide recalibration or controlled model updating, with subsequent validation before changes are implemented (9).

Together, the six articles in this Research Topic address only small parts of the ML model translation process from computer-to-bedside. They show that the barriers separating model development from clinical use are not confined to a single technical problem that can be solved through better algorithms. Translation can fail because the wrong problem was selected, because a model does not generalise, because it cannot be incorporated into clinical workflow, because intended users do not trust or adopt it, or because its output has no clearly defined role in decision-making. Progress in clinical ML will therefore depend less on producing additional retrospective models and more on designing research programmes that address these questions from the outset.

## References

[1] Deng J Elghobashy ME Zang K Patel SK Guo E Heybati K. So you’ve got a high AUC, now what? An overview of important considerations when bringing machine-learning models from computer-to-bedside. Med Decis Making. (2025) 45(6):640–53. 10.1177/0272989X251343082 40439482 PMC12260203

[2] Arshi B Wynants L Rijnhart E Reeve K Cowley LE Smits LJ. Number of publications on new clinical prediction models: a bibliometric review. JMIR Med Inform. (2025) 13:e62710. 10.2196/62710 40614260 PMC12252138

[3] Kolasa K Admassu B Hołownia-Voloskova M Kędzior KJ Poirrier J-E Perni S. Systematic reviews of machine learning in healthcare: a literature review. Expert Rev Pharmacoecon Outcomes Res. (2024) 24:63–115. 10.1080/14737167.2023.2279107 37955147

[4] Han R Acosta JN Shakeri Z Ioannidis JPA Topol EJ Rajpurkar P. Randomised controlled trials evaluating artificial intelligence in clinical practice: a scoping review. Lancet Digit Health. (2024) 6:e367–73. 10.1016/S2589-7500(24)00047-5 38670745 PMC11068159

[5] Saelmans A Seinen T Pera V Markus AF Fridgeirsson E John LH. Implementation and updating of clinical prediction models: a systematic review. Mayo Clin Proc Digit Health. (2025) 3:100228. 10.1016/j.mcpdig.2025.100228 40599890 PMC12212251

[6] Vasey B Nagendran M Campbell B Clifton DA Collins GS Denaxas S. Reporting guideline for the early-stage clinical evaluation of decision support systems driven by artificial intelligence: DECIDE-AI. Nat Med. (2022) 28:924–33. 10.1038/s41591-022-01772-9 35585198

[7] Preti LM Ardito V Compagni A Petracca F Cappellaro G. Implementation of machine learning applications in health care organizations: systematic review of empirical studies. J Med Internet Res. (2024) 26:e55897. 10.2196/55897 39586084 PMC11629039

[8] Hong JC Eclov NCW Stephens SJ Mowery YM Palta M. Implementation of machine learning in the clinic: challenges and lessons in prospective deployment from the system for high intensity EvaLuation during radiation therapy (SHIELD-RT) randomized controlled study. BMC Bioinformatics. (2022) 23:408. 10.1186/s12859-022-04940-3 36180836 PMC9526253

[9] Jenkins DA Martin GP Sperrin M Riley RD Debray TPA Collins GS. Continual updating and monitoring of clinical prediction models: time for dynamic prediction systems? Diagn Progn Res. (2021) 5:1. 10.1186/s41512-020-00090-3 33431065 PMC7797885

